# Energy-Efficient Massive Data Dissemination through Vehicle Mobility in Smart Cities

**DOI:** 10.3390/s19214735

**Published:** 2019-10-31

**Authors:** Salman Naseer, William Liu, Nurul I Sarkar

**Affiliations:** 1 Department of Information Technology and Software Engineering, Auckland University of Technology, Auckland 1010, New Zealand; william.liu@aut.ac.nz (W.L.); nurul.sarkar@aut.ac.nz (N.I.S.); 2 Department of Information Technology, University of the Punjab Gujranwala Campus, Gujranwala 52250, Pakistan

**Keywords:** smart city, delay tolerant network, infrastructure offloading, opportunistic network, vehicular mobility, energy consumption, carbon emission

## Abstract

One of the main challenges of operating a smart city (SC) is collecting the massive data generated from multiple data sources (DSs) and transmitting them to the control units (CUs) for further data processing and analysis. These ever-increasing data demands require not only more and more capacity of the transmission channels but also results in resource over-provision to meet the resilience requirements, thus the unavoidable waste as a result of the data fluctuations throughout the day. In addition, the high energy consumption (EC) and carbon discharge from these data transmissions posing serious issues to the environment we live in. Therefore, to overcome the issues of intensive EC and carbon emission (CE) of massive data dissemination in SCs, we propose an energy-efficient and carbon reduction approach by using the daily mobility of the existing vehicles as an alternative communications channel to accommodate the data dissemination in SCs. To illustrate the effectiveness and efficiency of our approach, we take the Auckland City in New Zealand as an example, assuming massive data generated by various sources geographically scattered throughout the Auckland region, to the control centres located in the city. Results obtained show that our proposed approach can provide up to four times faster transferring the large volume of data by using the existing daily vehicles’ mobility, than the conventional transmission network. Moreover, our proposed approach offers about 32% less EC and CE than that of conventional network transmission approach.

## 1. Introduction

In the near future, SCs are envisioned to provide services such as road lights conversing with the smart grids, urban parks associating with administrations, seasides conveying cautions on pollution levels, and flood alerts to disaster management. This results in generation of huge data, and they are stored in the data clouds or some data control centers for processing and analysis. A huge number of sensors, installed in each city will continually generate a tremendous amount of data [1]. For instance, Westminster City Council has introduced solar waste bins that can speak to city council workers and inform them how much full they are. The framework used infrared and telemetry sensors and prompted a 60% decrease in cost for waste collection. Smart homes are now turning into reality, and it is expected that they will be available commercially by next year. Splunk predicts that one smart home today can create as much as 1 GB of data in a week. This means that all the UK smart homes may generate more than 26 million GBs of information [2]. Furthermore, video surveillance of entire city, smart sensors, and smart girds also generate big volume of data on each day for processing and analysis [3].

The transmission of a large amount of SCs data is going to swamp existing infrastructure networks, and it presents some unwieldy challenges. The operator’s concern is to enhance the performance of their network by adding more capacity to their networks or by efficiently using the existing network resources. To address the issues of this explosion of data traffic we have different solutions. One of the expensive solutions is to elevate the existing networks to the next generation networks. Another expensive solution is to enhance the network capacity. However, the problem with these solutions is that it requires an enormous amount of cost for operational expense (OPEX) and capital expenditure (CAPEX) [4].

To transmit huge information in SCs, one of the candidate network is power line communication (PLC), it may provide LAN connectivity, WAN access, and some command and control capabilities [5]. However, interoperability problems, low dispersion and short-range communication of PLC networks become weak points for its success in the market of data transmission system [6]. Other energy-efficient solutions may include use of Zig-Bee, Bluetooth and WiFi along with PLC for smart metering and smart homes. Due to capacity and wireless range limitations, these solutions may be suitable for a short-range communication rather than long-distance communications [7]. Furthermore, fiber-optic in SC for huge data volume dissemination might be a good competitor. However, the city-wide deployment of optical-fiber system requires high budget [8].

**Figure 1:**
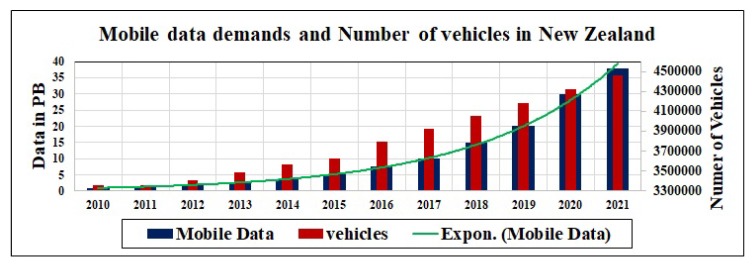
Mobile data demands and number of vehicles in New Zealand (NZ).

Lately, Cellular Networks (CNs) appear to be an alternative solution for big data communication in SCs. However, huge mobile data demands have already created the issues of devastating CNs [9]. Figure 1 shows that mobile data demands will grow up to 38 PB per month by 2021 in New Zealand, which is 380% more than 2010 [10]. Broadband Internet and traditional core networks could also be possible candidates for SC data transmissions, but these networks are also now congested networks, traffic on the Internet has increased more rapidly than its existing capacity [11,12]. As a result, the problem of data dissemination between data sources and control units in SC ought to be solved by using some other types of hybrid networks instead of the using only Wi-Fi, 3G, LTE, Internet and so forth [13]. Under this topic, vehicular networks by using the existing routine rides in the city could be a possible solution to disseminate big data in SCs.

On the other hand there is one more big challenge in energy consumption in information communication technology (ICT). Andrey gives his analysis on global EC, with the best average and worst-case scenarios. International Energy Agency (IEA) estimated that Global electricity consumption would grow by 2.8 to 3.4% per year and ICT EC will be a big ratio (51%) of global EC in 2030. The electricity demand of communication technology is increasing and will be 30,715 TWh of a worst-case scenario in 2030. Andrey predicts that depending on different scenarios of ICT, EC in 2010 is 8% to 14%, in 2020 6% to 21% and in 2030 8% to 51%, respectively. Hence, it is also a critical issue that needs to address [14,15].

These factors of congested networks and EC in ICT create interest in alternate energy-efficient solutions to reduce the pressure of data traffic on traditional core networks. In this direction, Cho and Gupta [16] enhanced the data transfer performance by suggesting the concurrent use of conventional network and postal system. Some portion of the data is forwarded using the conventional network while some other delay-tolerant data is forwarded on hard disks through the postal system and it requires comprehensive data scheduling. Munjal et al. proposed a centralized Software-Defined Vehicular Connectivity procedure that enables scalable and adaptive control of the scheduled vehicles to offload traffic [17].

Thus, it is useful to introduce an energy-efficient option by using the existing vehicular networks to address this problem of big data transmission and to reduce the load on existing infrastructure networks. In this paper, we are proposing to use daily routine rides of SC’s vehicles and annual average daily traffic (AADT) of Auckland city for delay-tolerant data transmission. The main contributions of this paper are as follows:

- We conduct a study to observe the correlation of current and predicted data demands of NZ’s main cities on system model.
- We develop a mathematical model to measure the degree of data offloading by considering the Poisson arrival of smart vehicles and RSUs of the road network.
- A case of Auckland City is evaluated by using annual average daily traffic (AADT) of Auckland City in our proposed model to reduce the end-to-end delay for big data transmission.
- We design new algorithms using multi-commodity flow problem to select an energy-efficient network for data transmissions.

The rest of the paper is organized as follows. A correlation study is reported in Section 2. Section 3 present the related work. The system overview is presented in section IV along with mathematical models. We present our network flow model used to minimize EC and CE in Section 4. Analysis is performed in Section 5, along with two case scenarios. Finally, the paper is concluded in Section 6 along with future work.

## 2. A Possible Alternate Channel

**Figure 2:**
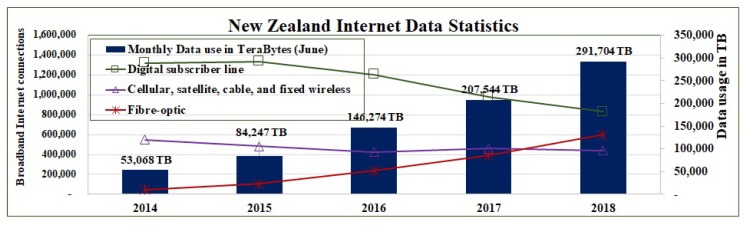
Past five years broadband data usage in NZ: Comparison of Fiber optic, Cellular/satellite/ and fixed wireless, DSL, and Monthly data use.

Like rest of the world, the data demands in New Zealand are increasing each year, as depicted in Figure 2. There is an increase of 40% data demand in 2018 and an increase of 54% in the number of fiber connections for faster data communication, to make up 32% of all broadband connections. On the other hand, a significant decrease of 25% is shown on the slow Internet and dial-up connections. More than 70% of all Internet broadband connections with no data limits and cap. These data demands of broadband connections include the use of 281,615,000 GB of data that is equal to 90 million hours of streaming, or 10,700 years, high definition online shows of TV. Mobile phone users consumed 10,089,000 GB data in June 2018, which is up to 56% from 2017 [18]. The use of mobile data has been grown by 600% in the past four years and the expected growth for the next four years is 50% per year [10].

**Figure 3:**
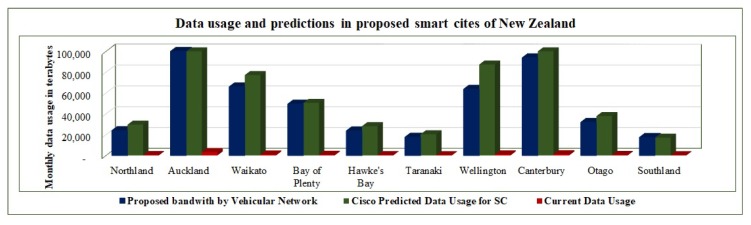
Data usage statistics of various NZ cities. Comparison of Cisco predicted, current, and the proposed data usage.

Cisco predicts that a SC with a populace of 1 million could produce 180 million GB data volume for each day or 42.3 ZB/month [19]. If we assume NZ main cities as smart cities, where data sources of different applications of smart cities like smart buildings, smart water, smart public services, smart lighting, smart mobility, smart waste management, smart meters, smart energy, smart sensors, and smart grids etc., will generate a massive volume of data and we need to transmit this big-data to data cloud, data centers or data control units. These expected data demands of 724 PB per day, will exceed the data usage beyond the available limit of existing infrastructure. There will be a massive gap between existing data usage and required data usage of these cities, as shown in Figure 3.

We propose a complimentary network, the vehicular network as a hybrid network, along with existing infrastructure, and mobile networks. Business Insider (BI), predicted that in 2021, 82% of all vehicles would be shipped as smart and connected vehicles [20]. These smart vehicles will have wireless interfaces, global positioning system, storage capacity, and processing capacity. We assume NZ’s main cities as SCs. Ministry of transport NZ predicted that NZ has 0.79 per capita vehicles in 2016. This value is keep on increasing with an average increase of 2.12% in per capita vehicles since the past five years [21] as shown in Table 1. Figure 3, also predicts the parallel growth in mobile data demands and vehicle volume in NZ. Hence, it is forecasted that this significant volume of connected vehicles can produce huge bandwidth on the road network of NZ’s smart cities. By using the data set of the ministry of transport NZ [21,22] as shown in Table 1, our proposed system predicts the vehicular network in SCs of NZ could produce a massive bandwidth up to 607 PB, if only 20% smart vehicles in NZ will transfer only one data assignment on a daily basis, as shown in Figure 3.

## 3. Related Work

Presently, different data clouds offer a data storage space on their web servers to their clients. Normally, numerous duplicates of data are stored on these servers [23]. The best example to transfer data from a client location to these servers is the data delivery services of Amazon Web Services (AWS). AWS offers data movement by using their data vehicles. For this purpose, AWS sends their vehicle to the client, the client loads required data onto the vehicle, and the vehicle is driven back to AWS for loading the client’s data onto their web servers [24]. A digital map provider company Digital Globe used snowmobile services of AWS to transfer 70PB data onto the cloud of AWS [25]. This type of data transmission requires some fiscal cost because it needs a dedicated vehicle for this purpose. Moreover, it consumes energy and emits carbon in the environment.

A big data of delay-tolerant applications can be transferred by efficiently using the energy of daily routine trips of vehicles. Vehicular delay-tolerant network (VDTN) [26], is built on the theory of delay-tolerant network (DTN). It handles delay-tolerant applications at low cost and on unpredictable network conditions. Vehicles can be used as data carriers between terminal nodes either in rural areas or in emergency scenarios. This strategy can be helpful in both V2V and V2I communication. In this direction, Kashihara et al. [27], proposed a data offloading scheme to offload data, particularly to scheduled vehicles by using short-range and high-speed wireless communication. The motivation was to reduce traffic congestion because of the high data volume on traditional networks. Similarly, Hunjet et al. [28] and Usbeck et al. [29] uses VDTN and implement data forwarding schemes by using data farriers. A vehicular data dissemination project is implemented in France to reduce load on conventional networks [30]. Dessler et al. [31] used parked vehicle for data communication among other vehicles and proposed a protocol called vehicle cord where parked vehicles are used as RSUs.

In another work [32], Cho et al. uses hybrid network to upgrade the performance by proposing the concurrent use of traditional core network and postal framework. A portion of the time-critical data is exchanged by using the conventional core network while postal system is used to transfer delay-tolerant data with the help of hard drives. It requires complex data scheduling for data forwarding. Marincic et al. upgrade a similar work to minimize the EC and reduce the CE [33].

Moving Vehicles on the road and roadside APs of WiFi Network can also be used as a practical solution for cellular data offloading in SCs, called vehicular Wi-Fi offloading. The research in this direction has an objective to enhance the cellular data offloading performance, particularly for delay-tolerant and non-interactive applications [34,35]. At the same time, the advantages of opportunistic communication and D2D revolution are sound for some vehicular related use cases. It can empower location-based peer-to-peer applications and services. For example, thinking about the huge number of connected smart vehicles, a new software update of the smart vehicles can put a critical load on the cellular network, and cost huge money for vehicles owners. In this way, the new update can be downloaded by some designated vehicles by using D2D communication with RSUs, and then this update can be exchanged to other vehicles by D2D transmission. Along these lines, the majority of the cellular load can be shifted to V-D2D communication and accordingly cellular bandwidth, energy, and cost can be saved [36].

A network architecture is explained in [12] for smart city data offloading by using smart vehicles, and numerical analysis of this proposed architecture is demonstrated in [37]. In this architecture, the authors apply D2D communication, the daily vehicle count of Auckland roads and calculate the delay, throughput, and energy consumption. The proposed system outperforms the Internet with dedicated links of 100 Mbps and 1 Gbps. Also, the system can offload traffic by consuming less energy than the Internet. To transfer 20 TB data from a data location to the control unit, it consumes 40% less energy as compared to the traditional core network. In this paper, we are trying to describe how much data can be uploaded/downloaded to/from RSU while vehicle is on the move and how proposed algorithm can be used to select an energy-efficient network mode to transfer big data between two points.

## 4. The System Model

**Figure 4:**
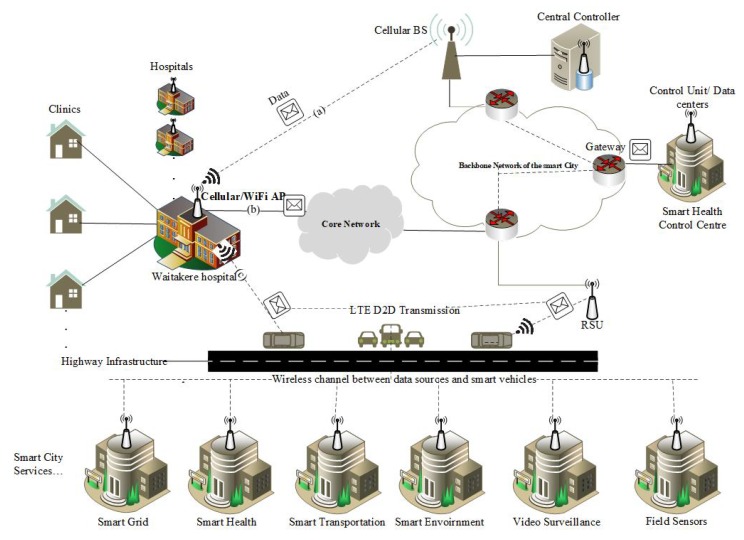
System model overview.

The proposed system model is shown in Figure 4. The wireless coverage of cellular network is available everywhere in the city, and smart vehicles are moving on the roads. These smart vehicles have GPS, WiFi, and cellular interfaces, also have storage and processing capacity. They can form a network with base station (BS) by using cellular interface, with roadside units (RSU) or with other vehicles by using WiFi interface. A central controller (CC) server is installed at cellular network. This controller will govern overall communication between data sources and control units.

For our proposed work, we assume that various SC devices and sensors generate huge volume of data in a city and these devices are the data sources. We need to transfer this data to different destinations in this case CUs are the destinations. CC selects cellular network or traditional core network to forward delay-sensitive data. For delay-tolerant data, CC selects cellular network, core network, or proposed vehicular network. Energy-efficient network mode selection algorithm is placed on CC server. When an information source needs to send information to a destination CU, it sends an information request packet to CC on cellular control channel. CC will choose the most appropriate alternative, to exchange information by means of core network, cellular network or vehicular network. This decision is made by considering the information of vehicle count on the road, delay-tolerant interval, history of vehicle’s trajectory and energy cost. Based on these results, CC selects the suitable network, if infrastructure network is more appropriate for the given set of demands then CC guides the source to send data on path (a) or (b) of Figure 4, otherwise data can be forwarded by using vehicular transport network on path (c). For each given set of data transfer requirements, CC chooses optimal vehicles by using their trajectory’s history and informs the DS to send data by using these particular vehicles.

On the receiver side, these vehicles upload the data whenever they encounter with an RSU by using their wireless interface. The RSUs are connected to the backbone network of the city and send the data packets to CU. After receiving the data, CU send the acknowledgments to the CC server. If a particular data packet is not transmitted to the CU before the delay-tolerant indicator expires, then CC informs to particular data source for re-transmission of missing data packets, by using the conventional network.

To calculate the delay and EC, following are the models, that are used in this paper for mathematical calculations. Notations used in the mathematical model are listed in Table 2.

### 4.1. Delay Model

To transfer the data from source to destination, following are the two models of end-to-end delay for transport network and core network.

#### 4.1.1. Delay Model for Transport Network

Effective distance: We have to identify the suitable region for vehicle in the wireless coverage area of an RSU. Normally, the packet loss rate depends upon the received power. By increasing the received power we can reduce the packet loss rate. Received power also depends upon the distance from the RSU. Therefore, to calculate the packet loss rate we can use the distance from RSU. Hence, to reduce the packet loss, it is essential to keep a vehicle in the appropriate coverage area of the RSU. We can calculate the path loss PL(d) at a distance *d* by using path loss formula.

(1) $$PL\left(d\right)\left[dB\right]=PL_{F}\left(d_{0}\right)+10n\log\left(\frac{d}{d_{0}}\right)+\Psi$$

where $d_{0}$ is the reference distance where path loss inherits the characteristics of free-space loss $PL_{F}$, *n* is path loss exponent, depending upon the propagation environment. Ψ is the Gaussian random variable.

From Equation (1) we can find the effective distance from the vehicle to RSU.

(2) $$d=d_{0}\times 10^{\frac{PL_{F}\left(d_{0}\right)+\Psi -PL}{10(n)}}$$

*d* is the distance from the RSU to the vehicle and effective distance can be 2d, the diameter of the wireless coverage area of RSU. In other words, each vehicle can have a chance to transmit the data packets to RSU at a distance from *0 to 2d* meters. In fact, the effective distance is restricted by each road, and it depends upon vehicle density on the road. Vehicle density is very high in urban areas and low in rural areas.

Delivery probability with vehicle density: The other parameters that affect the effective region of RSU are delay and bandwidth of WiFi is a key factor that can control the delay. RSU has a fixed bandwidth that can be shared among all vehicles inside the coverage area. If vehicle volume on the road is greater, then smaller will be the bandwidth share for vehicles. We assume a fixed number of vehicle *m* in the coverage area of RSU with bandwidth $B_{RSU}$. As we know that vehicles that connect to RSU follow Poisson arrival process {f(t), t ≥ 0} with parameter λ> 0 and vehicle volume on the road varies at different intervals of the day [38]. We can convert a day into various intervals, according to vehicle volume on the road, and these intervals follow the Poisson distribution. Suppose that there are *n* intervals ($I_{1}$,$I_{1}$ …$I_{n}$) with parameters ($\lambda_{1}$, $\lambda_{2}$ …$\lambda_{n}$). The probability of vehicles in the coverage area of an RSU at any time *t* can be calculated as follows.

(3) $$P\left\{f\left(t+i\right)-f\left(i\right)\right\}=e^{-\lambda t}\frac{{\left(\lambda t\right)}^{m}}{m!}$$

where *i* is the start time of each interval, (t+i) ∈ ($I_{1}$,$I_{1}$…$I_{n}$), and λ∈ ($\lambda_{1}$, $\lambda_{2}$…$\lambda_{n}$).

The vehicle density in the effective region of an access point depends upon the average speed $\bar{s}$ and Poisson arrival parameter lambda λ. By using different value of lambda ($\lambda_{1}$, $\lambda_{2}$…$\lambda_{n}$) in different intervals ($I_{1}$,$I_{1}$…$I_{n}$), we calculate the vehicle density ρ in the effective distance *d* of access point.

(4) $$\rho =\frac{\lambda }{\pi \bar{s}d}$$

Suppose $B_{RSU}$ is the bandwidth of RSU and $B_{v}$ is the shared bandwidth of each vehicle when there are *m* vehicle in the coverage of RSU then

(5) $$m=\frac{B_{RSU}}{B_{v}}$$

Transmission probability Φ, with *m* number of vehicles can be calculated as follows.

(6) $$\Phi =\sum_{v=1}^{m}\left(e^{-\lambda t}\frac{{\left(\lambda t\right)}^{v}}{v!}\right)$$

Degree of data offloading: By using (2),(3), and (6) we can calculate the data transmitted/offloaded by a vehicle to RSU as follows.

(7) $$B_{v}=B_{RSU}\times \Phi \times \frac{d}{\bar{s}}$$

Equation (7) can be represented in term of vehicle density (4) as follows.

(8) $$B_{v}=B_{RSU}\times \Phi \times \frac{d}{\frac{\lambda }{\pi \rho d}}$$

where *d* is the distance of the vehicle from RSU and $\bar{s}$ is the speed of the vehicle.

End-to-end delay: The time $T_{d}$ required to transfer a big data $D_{vol}$ from source A to destination B depends upon two things. First travel time between A and B $T_{AB}$, 2nd the data loading time $T_{L}$.

(9) $$T_{d}=T_{AB}+T_{L}$$

If $d_{AB}$ is the distance between these two points and $\bar{s}$ is the average speed then $T_{AB}$ can be calculated as follows.

(10) $$T_{AB}=\frac{d_{AB}}{\bar{s}}$$

If $N_{v}$ is the total number of vehicle required to transfer data $D_{vol}$ then data loading time $T_{L}$ can be calculated as follows.

(11) $$T_{L}=\frac{D_{vol}}{N_{v}}$$

$N_{v}$ depends upon vehicle count $V_{c}$ on the road, bandwidth share of each vehicle $B_{v}$ and probability μ of the drivers or vehicles willing to participate in this proposed system, then $N_{v}$ can be calculated as follows.

(12) $$N_{v}=V_{c}\times B_{v}\times \mu$$

By applying the value of $N_{v}$, $T_{AB}$ and $T_{L}$ in (9).

(13) $$T_{d}=\frac{d_{AB}}{\bar{s}}+\frac{D_{vol}}{V_{c}\times B_{v}\times \mu }$$

By using the value of $B_{v}$ from (7).

(14) $$T_{d}=\frac{d_{AB}}{\bar{s}}+\frac{D_{Vol}}{V_{c}\times B_{RSU}\times \Phi \times \frac{d}{\bar{s}}\times \mu }$$

#### 4.1.2. Delay Model for Traditional Core Network

For core network case, the total delay depends upon the number of packets and number of intermediate nodes. Suppose *L* is the length of each packet then the total number of packets *N* can be calculated as follows.

(15) $$N=\frac{D_{Vol}}{L}$$

Let Tp be the processing delay, Tq be the queuing delay, and Tt be the transmission delay and $N_{Nodes}$ is the total number of nodes between sender and receiver then total end to end delay can be calculated as follows.

(16) $$T_{Core}=N_{Nodes}\times 1^{st}PacketDelay+\left(N-1\right)\times \left(T_{p}+T_{q}+T_{t}\right)$$

We assume in case of a dedicated link when a packet arrives at a node; it will find no packet ahead in the queue. Then queuing delay becomes zero. In such case parallel processing will be performed at each node then the final end to end delay can be calculated as follows.

(17) $$T_{Core}=N_{Nodes}\times 1^{st}PacketDelay+\left(N-1\right)\times T_{t}$$

If *B* is the transmission bandwidth then Equation (17) can be written as.

(18) $$T_{Core}=N_{Nodes}\times 1^{st}PacketDelay+\left(N-1\right)\times \frac{L}{B}$$

### 4.2. Energy Model

To transfer the data from source to destination, following are the two models of energy consumption for transport network and traditional core network.

#### 4.2.1. Energy Model for Transport Network

EC for delay-tolerant information transporting between two points by using vehicles depends on two things. 1*^st^* EC for data loading and 2*^nd^* EC for transporting the weight of storage device from point A to B.

(19) $$E_{Veh}=E_{DataLoad}+E_{Transport}$$

If *k* is the total number of offloading points between A and B then $E_{DataLoad}$ can be calculated as follows

(20) $$E_{DataLoad}=2\times \sum_{i=1}^{k}E_{D2D_{i}}$$

$E_{transport}$ depends upon the distance between the sender and receiver, gross weight of the vehicle, and fuel economy of the vehicle. If *n* vehicles are used to transport total data between them and $W_{Storage}$ is the storage device weight then we can calculate the EC for whole data shipment as follows.

(21) $$E_{Transport}=C_{fuel}\times W_{Storage}\times \sum_{i=1}^{n}E_{Shipment_{i}}$$

$C_{fuel}$ is fuel constant, and it converts the volume of fuel into energy consumed i.e., from litters to joules. If FE is the fuel economy then $E_{Shipment}$ can be calculated as follows.

(22) $$E_{Shipment}=\frac{d_{AB}}{FE\times V_{Load}}$$

If $W_{Total}$ is the gross weight of the fully-loaded vehicle then $V_{Load}$ can be calculated as follows.

(23) $$V_{Load}=W_{Total}-W_{Empty}$$

#### 4.2.2. Energy Model for Core Network

Data transmission begins from the 1st datagram at the source and ends when the last datagram is delivered at the destination. EC for conventional network depends upon the energy consumed at the sender, receiver, and all intermediate devices. These devices contain switches and routers. If $D_{Vol}$ is the total data volume, $E_{SR}$ is the EC at source and destination, and we consider the incremental EC at all *k* intermediate devices, then the EC for conventional network case can be calculated by using the following equation.

(24) $$CnEc=E_{SR}+\sum_{i=1}^{k}E_{inc_{i}}$$

If $B_{up}$ is the uploading and $B_{down}$ is downloading bandwidth at sender and receiver respectively, ▵P is power change at sender/receiver while transmitting/receiving the data, then $E_{SR}$ can be calculated as follows.

(25) $$E_{SR}=max\left(\frac{D_{Vol}}{B_{up}},\frac{D_{Vol}}{B_{down}}\right)\left(▵P_{Sender}+▵P_{Receiver}\right)$$

For intermediate devices, if $E_{bit}$ is the energy cost per bit then energy consumption for total $D_{Vol}$ data is calculated as follows.

(26) $$E_{inc}=D_{Vol}\times E_{bit}$$

Energy consumed per bit is calculated as a fraction of the max power $P_{max}$ and available bandwidth *B*.

(27) $$E_{bit}=\frac{P_{max}}{B}$$

## 5. Minimizing Energy Cost

The goal of this work is to minimize the total cost of sending the data requirements across the complex road network or traditional core network, while satisfying all demands/supplies, and respecting arc capacities. We solve the energy optimization problem for road network by using multi-commodity flow problem and calculate the energy cost for optimal paths. Similarly we calculate the energy cost for core network and finally we find that which network is suitable for data transmission with respect to energy consumption.

### 5.1. Multi-Commodity Flow Problem

Let G=(V,E,C,A) be a capacitated undirected graph, where *V* is set of data sources or data centers locations, *E* is set of road links between data sources and destinations, *C* is the capacity of each road w.r.t vehicle count, and *A* is a set of cost per unit flow for a commodity $b_{i}$ on each link (i,j)∈E. $R=\{\left(s_{i},t_{i},b_{i}\right)\}$ be a set of requirements, $s_{i}\in V$ is data source and $t_{i}\in V$ is destination data center for commodity $b_{i}$. For each edge (i,j)∈E and each commodity *r*, associates a cost per unit of flow, designated by $a_{ij}^{r}$. The demand (or supply) at each node i∈V for commodity *r* is designated as $b_{i}^{r}$, where $b_{i}^{r}>0$ denotes a supply node and $b_{i}^{r}<0$ denotes a demand node. We define decision variables $x_{ij}^{r}$ that denote the amount of commodity *r* need to send from node *i* to node *j*. The amount of total flow, for all commodities, that can be sent across each link is bounded above by $c_{ij}$. We need to minimize the transport network energy consumption TnEc by using the vehicle mobility of complex road network.

**Minimize**:

$$TnEc=\sum_{(i,j)\in A}\sum_{r\in R}a_{ij}^{r}.x_{ij}^{r}$$

**Subject to**:

$$\sum_{r\in R}x_{ij}^{r}\leq c_{ij}\;\left(i,j\right)\in E\;\left(Capacity\right)$$

$$\sum_{i,j\in E}x_{ij}^{r}-\sum_{i,j\in E}x_{ji}^{r}=b_{i}^{r}\;i,j\in V,r\in R\;\left(Balance\right)$$

$$x_{i,j}^{r}\geq 0\;\left(i,j\right)\in E,r\in R$$

$$\;\sum_{i,j\in E}x_{ij}^{r}\left(n\right)-\sum_{i,j\in E}x_{ji}^{r}\left(n\right)=\left\{\begin{array}{cc} \;\;\;b_{i}^{r} & if\;n=s^{r}\;\\ \;-b_{i}^{r} & if\;n=t^{r}\;\\ \;\;\;0 & otherwise \end{array}\right.\;\left(Flow\;conservation\right)$$

∀n∈Vandr∈R

We use Algorithm 1, to solve the above energy optimization problem of multi-commodity flow, for multiple commodities of data transmission across the road network and calculate the energy-efficient paths.

**Algorithm 1:** Minimum cost multi-commodity flow

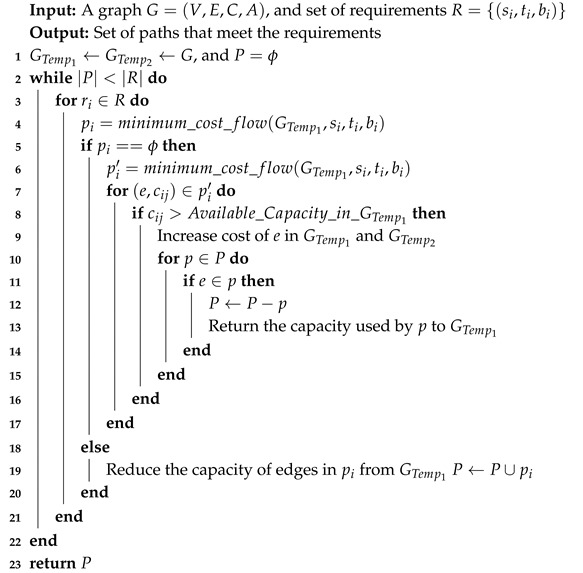



### 5.2. Energy-Efficient Network Mode Selection

Let $G^{{}^{\prime }}=\left(V^{{}^{\prime }},E^{{}^{\prime }},A^{{}^{\prime }}\right)$ be a capacitated undirected graph, where $V^{{}^{\prime }}$ is a set of intermediate nodes (routers and switches), $E^{{}^{\prime }}$ is a set of the links between these intermediate nodes, and $A^{{}^{\prime }}$ is a set of unit energy cost to transfer a data commodity $b_{i}$ on these links. We calculate the energy cost CnEc for data transfer between data source and data center by using traditional core network from Equation (24). For transport network energy cost TnEc can be calculated from Algorithm 1. The central controller apply Algorithm 2 to take the decision which network is suitable for a given set of data demands. This decision is forwarded to all data sources and intermediate nodes. Finally they select appropriate energy-efficient network interface to forward the data.

**Algorithm 2:** Energy-efficient network mode selection

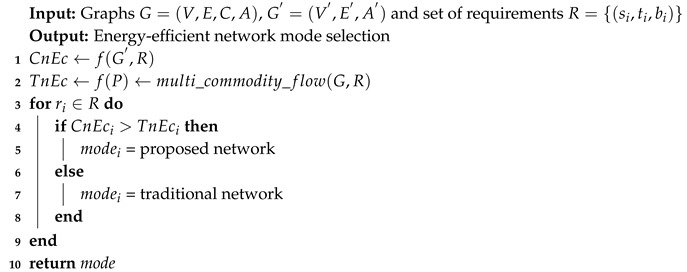



## 6. Performance Analysis

This section describes the numerical analysis of our proposed system with conventional network system. In first part, we set the value of various parameters and then we present two case scenarios to compare both systems.

### 6.1. Parameters Setting

To evaluate the proposed system model, we consider a straight expressway. Vehicles are moving with some predefined average speed $\bar{s}$ along the straight road. We are assuming that the vehicles are moving only in one direction for simplicity. We calculate the effective distance and data transfer probability in the wireless coverage area of an RSU. We also measure the vehicle density with different speeds of vehicles. Finally, by applying these parameters, we calculate the degree of data offloading by using different speeds and vehicle density.

**Figure 5:**
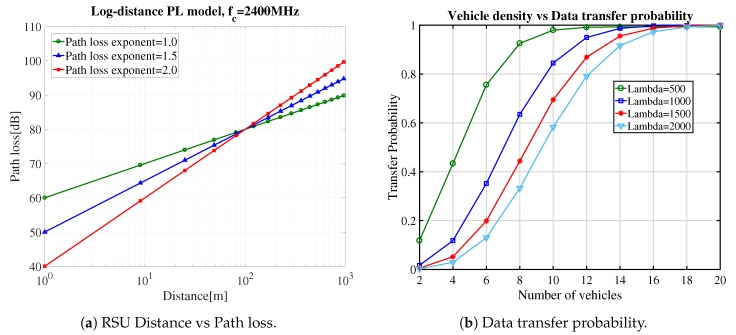
Effective distance and Delivery probability.

Effective distance: To calculate the effective distance, we set reference distance $d_{0}=100$ m and carrier $f_{c}=2.4$ GHz and apply the different path loss exponent values, according to Equation (2). The relationship between path loss [dB] and distance is shown in Figure 5a. Path loss increases with the distance between vehicle and RSU and approaches to 80 dB for 50 to 100 m. For the remaining analysis we set effective distance *d* = 100 m.

Delivery probability: The fixed bandwidth of RSU is divided among all the vehicles in the wireless coverage area of WiFi. Data transmission probability increases exponentially with different densities of vehicles as shown in Figure 5b. We vary the Poisson arrival parameter λ from 500 to 2000 by using Equation (6). The trend shows that the data transfer probability reaches to 0.95 in all cases when the number of vehicles approaches to 15.

**Figure 6:**
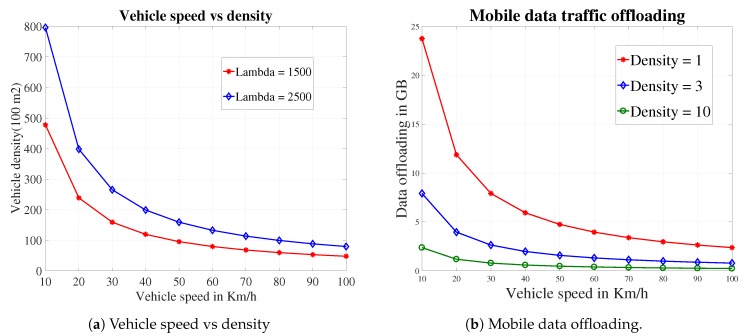
Results for degree of data offloading.

Vehicle density: ρ is evaluated with different speeds of vehicles by using effective distance *d* and Poisson arrival parameter λ in Equation (4). We vary the value of λ from 500 to 2500 and set effective distance *d* = 100. Vehicle speed is used to get the vehicle density in a specific area as shown in Figure 6a. If we increase the vehicle speed, then vehicle density decreases exponentially.

Degree of data offloading: By considering the above evaluation, the data offloading can be calculated by using Equation (7) in the wireless coverage area of an RSU. According to Figure 5a, we set the effective distance of the vehicle from RSU d=100, and we set the transmission probability ρ = 0.95 according to Figure 5b. A vehicle can transfer various amount of data to RSU by using different speeds. The time in which a vehicle stayed connected with an RSU is inversely proportional to vehicle speed. According to Equation (7), the data transfer rate varies with the different speeds of vehicle and connection time duration of a vehicle decreases with increase in the speed of the vehicle. Moreover, Figure 6b, shows that if we increase the vehicle density, i.e., there are more vehicles that want to send the data then the bandwidth will be divided among all vehicles.

In the following two subsections, we present two case scenarios for comparing the performance in terms of delay and EC.

### 6.2. Case Scenario I—Auckland City Case Scenario for Delay Tolerant Study

**Figure 7:**
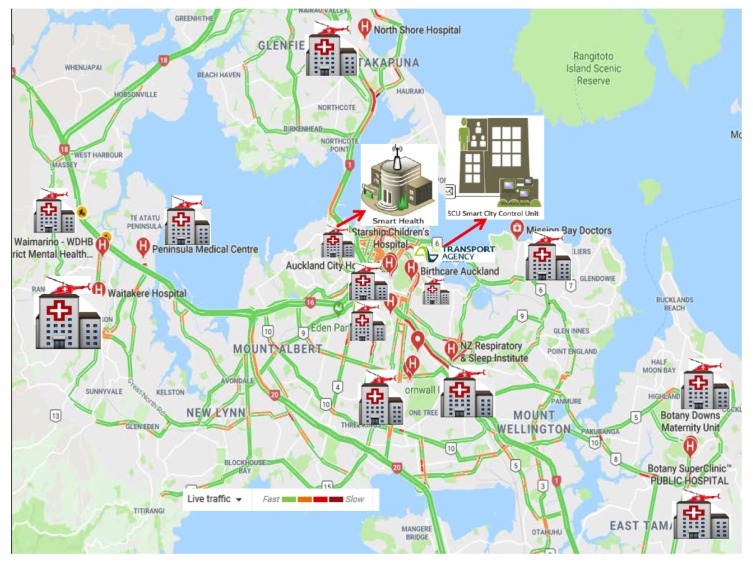
Auckland City Case Scenario for Delay Tolerant Study.

**Figure 8:**
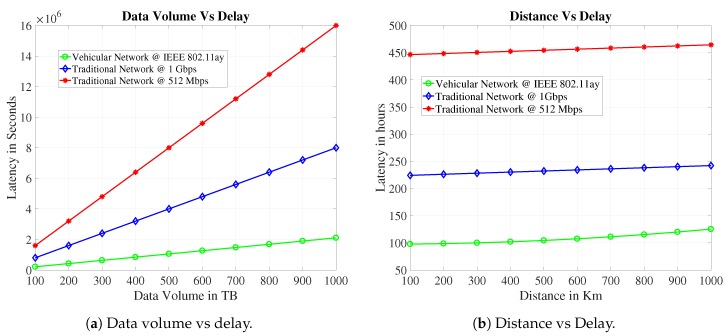
Delay performance study for Auckland City case scenario.

The proposed case of Auckland City is shown in Figure 7. We propose two cases for delay-tolerant information transmission, the core network, and the vehicle transport network. Assume a source at SH16 Royal Rd Off Ramp to Hobsonville Rd/SH18 Off Ramp WB with reference ID:01610016 in Auckland City needs to send some delay-tolerant data $D_{Vol}$ to a destination in the city center. For the vehicular system, we find the distance *d* between these two points is 23 Km by using Google maps and vehicle count AADT on the said link is 27857 [39]. The average speed $\bar{s}$ of the vehicle on this link is 50 Km/h. Assume each vehicle has IEEE 802.11ay wireless interface and disk capacity is 256 GB. We assume that if only 20% (μ=0.2) drivers take part in this proposed framework, then by applying Equation (14) we can estimate the delay value of the proposed network. In the core network case, a cellular network, a wired network, or a wireless network can be used for data forwarding, and its delay can be estimated by using the Equation (18). We exchange the different information volume between these two locations and get the outcomes as appeared in Figure 8a. For comparisons, we use the bandwidth 512 Mbps and 1 Gbps in traditional core network case. The outcome demonstrates that our proposed vehicular network outperforms the traditional core network. We enhance the data size and look at its impact on delay for data transmission. The results demonstrate that for huge information volume, the delay increases significantly for core networks as compared to our proposed vehicular network.

In the next case as shown in Figure 8b, we compare data transmission delay with distance for both cases. We transfer 100 TB data between two points by varying the distances. In this case, more routers and intermediate nodes will be used in core network, and delay depends upon the distance between two points as well. This transmission delay depends upon queuing delay, propagation delay, transmission delay, and processing delay. We assume that there is no queuing delay on the core network when a packet arrives in at the router, there is no inbound and outbound queue. Also, assume that the core network is built on fiber. Hence the propagation delay can also be ignored. We consider only transmission and processing delay and assume that there is a router after every 100 Km. Vehicular network delay also increases as the distance increases to the destination because each vehicle takes more time to reach the destination when we increase the distance. Similarly Figure 8b, also shows that the delay in the case of core networks also increases as the number of routing devices keeps on increasing. The results show that our system outperforms the traditional core networks when we increase the distance.

**Figure 9:**
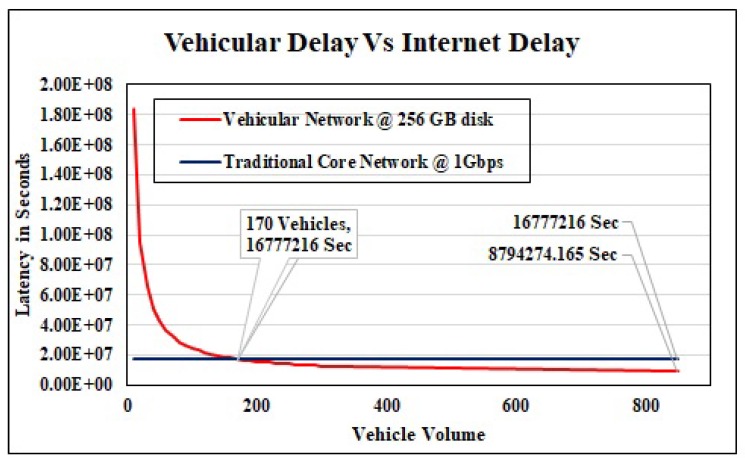
Latency in Transport Network Vs Traditional Network.

Vehicle Volume: Equation (14) shows that throughput of the vehicular network also depends upon vehicle volume on the road. To get the impact of vehicle count, we forward a massive size of data 1 PB between the same two points in Auckland. We vary the vehicle count on the road from 0 to 1000, By keeping constant the average speed and disk capacity, 50 Km/h and 256 GB respectively. Figure 9, demonstrates that at the beginning delay is very high in our proposed case as compared to traditional network, and this latency keeps on decreasing when we increase the vehicle volume on the road. In this scenario our system outperforms the conventional network with a dedicated bandwidth of 1 Gbps when vehicle volume is higher than 170. Our proposed scheme reduces the delay up to 47% when vehicle count is 850 in between these two points.

**Figure 10:**
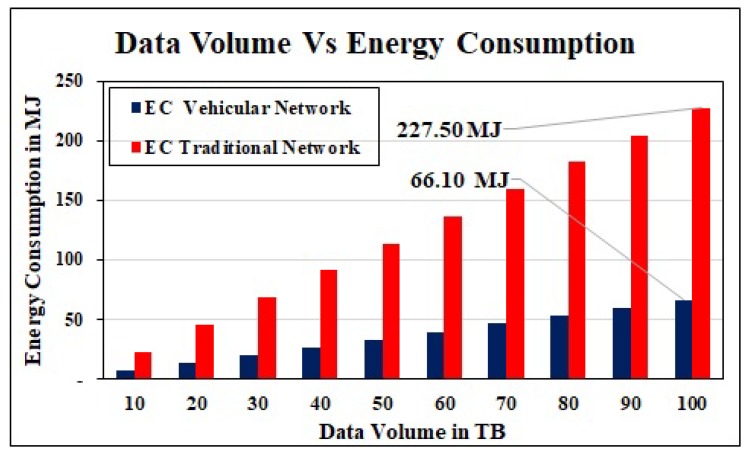
Data volume Vs Energy Consumption.

Energy Consumption: Our proposed EC model demonstrates that the EC relies on the information volume and the distance between sender and receiver. For core network, we assume that uploading and downloading bandwidth is 0.1 Gbps at sender and receiver. The intermediate nodes between sender and receiver are eleven LAN switches, two core and edge router. To get the impact of data volume, we forward different sizes of data between the same sender and receiver. The results in Figure 10, show that when we keep on increasing the data size, the EC also increases proportionally in both vehicular and conventional network cases. Vehicular system outperforms the traditional core network. It consumes only 66MJ energy to forward 100 tera byte data whereas the core network consumes 227 MJ of energy.

### 6.3. Case Scenario II—Finding the Best Routes

**Figure 11:**
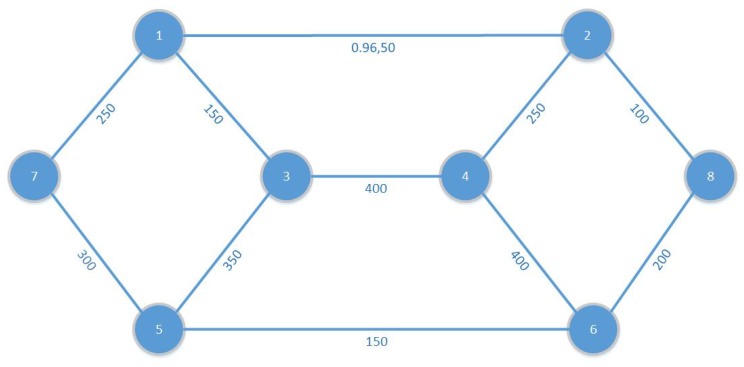
Graph of building network flow.

In this case scenario, we proposed a solution, where a road network is investigated for data transfer assignments as shown in Figure 11. Here, nodes represent the data offloading locations and links represent the roads. Each road has the traffic count as the capacity of the road. We use an embedded storage device of weight 0.95 Kg in the vehicle and calculate the energy cost to transfer 1TB of data for each road. In these calculations, we use Toyota Prius as a relaying vehicle, and the value of fuel constant is assumed as Cfuel = 37,624,722.29 J/L [40] in Equation (21). These calculations are presented in Table 3. For each offloading request; we apply our Algorithm 1 to find the best route by solving the data transfer assignment as a traditional multi-commodity flow problem.

The data set in Table 3, provides the cost $a_{ij}^{k}$ of sending a unit of commodity *r* along arc (i,j), with distance and speed of that particular road. To calculate the EC of the traditional core network we use Table 4.

#### 6.3.1. Energy Consumption

**Figure 12:**
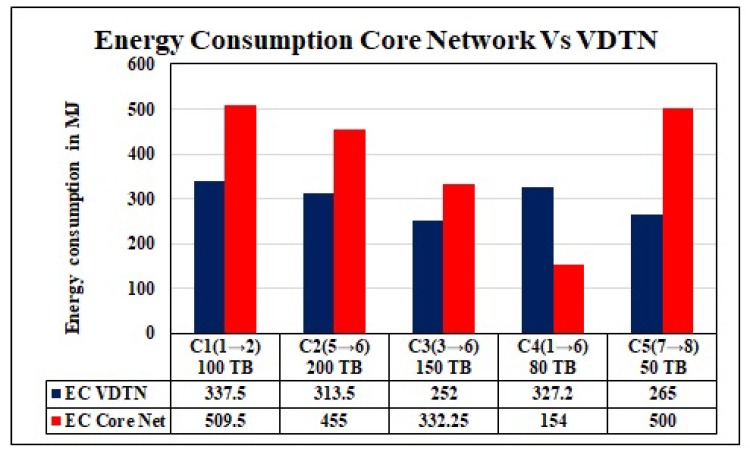
EC: A comparison of the core network and VDTN.

By using this multi-commodity flow model and our energy cost model, we evaluate the EC cost for a given set of five commodities (Figure 12) R={c1,c2,…,c5} of delay-tolerant data, with a total data flow of 580 TB. We execute Algorithm 1 on SaS optimization tool [41] to find a set *P* of energy-efficient paths and optimal solutions for a given set of commodities to transfer data by using road network. To calculate the EC of the traditional core network, we use graph $G^{{}^{\prime }}=\left(V^{{}^{\prime }},E^{{}^{\prime }},A^{{}^{\prime }}\right)$ by applying Equation (24), and unit cost for EC to transfer a data demand on each edge in MJ/TB is calculated in Table 4. Calculated results for the core network and VDTN are shown in Figure 12. It shows that our proposed data transport model outperforms the traditional core network, for all the commodities other than the commodity number 4. In this case the distance between the data source and destination is high. That is why vehicles consume more energy to transfer data at the final destination. Moreover, for this demand, a huge amount of energy is also consumed at intermediate nodes for data offloading from vehicle to intermediate nodes and uploading from intermediate nodes to vehicles. To transfer 580 TB data, the traditional core network consumes 1950 MJ energy, and our proposed vehicular network consumes 1495 MJ energy. In this way, our proposed vehicular network can save up to 24% of energy costs for these data assignments.

#### 6.3.2. Energy Efficient Network Mode Selection

On the basis of the above calculated energy costs for traditional core network CnEc and for transport network TnEc, the central controller apply Algorithm 2 to take the decision which network is suitable for each commodity in a given set of data demands. Central controller forward this decision to concerned data sources and intermediate nodes. The data sources and intermediate nodes select appropriate energy-efficient network interfaces to forward the data. With this optimal network mode selection, we can save the energy cost more than 32% for this given set of commodities.

#### 6.3.3. Carbon Emission

**Figure 13:**
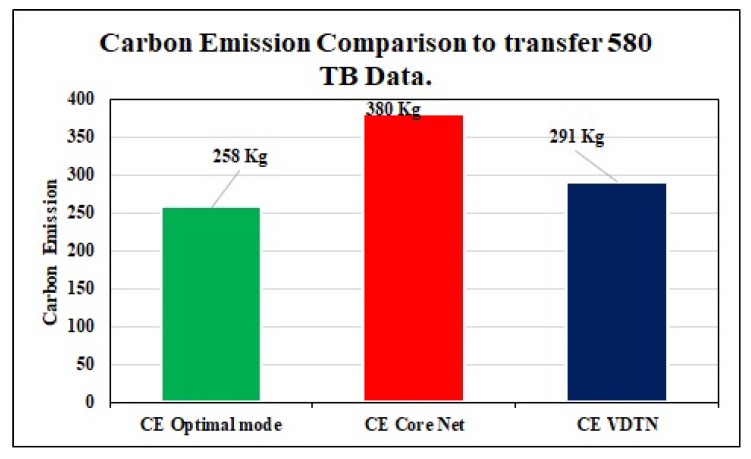
CE: A comparison of the core network, VDTN and optimal network mode.

By applying energy-efficient network mode selection, we can further reduce the energy cost and carbon consumption. Information exchange for both core network and VDTN consumes energy from various means like fuel and power. This usage of energy transmits carbon into the environment. Figure 13 demonstrates that the CE, is less for our proposed optimal model as compared to traditional core networks and VDTN. To evaluate these outcomes, we used the carbon emission 0.703 $CO_{2}$ Kg/kWh [42] and the conversion unit 1 MJ = 0.2777778 kWh [43].

## 7. Conclusions

In this paper, we developed both delay and energy models for sustainable data dissemination to be used to tackle typical SC daily data traffic. As a proof-of-concept of our algorithms, we presented Auckland City case scenarios where delay tolerant data is delivered to data centres. We found that our proposed system can effectively use the daily vehicle mobility of Auckland City for enormous information transmission to reduce the cost of EC and CE. The results obtained show that the proposed system can offer up to four times better data transfer rate than the dedicated core network with a data rate of 1 Gbps. For data transmission scenarios, our proposed approach can offer about 32% better EC and CE than the traditional network. The main conclusion is that our proposed system is suitable for delay-tolerant data delivery applications as it can reduce the network load further by sharing the burden of congested networks. Developing a more flexible and mature data offloading communication system by optimal mode selection on the basis of multi-objective optimization for delay-tolerant interval as well as energy cost, for data transmission is suggested as our future work.

## Figures and Tables

**Table 1:** Average percentage increase in per capita vehicles.

City Name	Population	Per Capita Vehicle	Number of Vehicles	Yearly %Age Increase
Northland	168,300	0.71	119,493	1.14
Auckland	1,569,900	0.71	111,4629	2.03
Waikato	439,100	0.75	329,325	1.37
Bay of Plenty	287,100	0.86	246,906	1.66
Gisborne	47,400	0.66	31,284	0.62
Hawke’s Bay	160,000	0.74	118,400	1.64
Taranaki	115,700	0.76	87,932	0.8
Wellington	496,900	0.64	318,016	1.28
West Coast	32,700	0.90	29,430	1.61
Canterbury	586,400	0.80	469,120	0.25
Otago	215,000	0.74	159,100	1.38
Southland	97,300	0.89	86,597	1.37

**Table 2:** Notations used in this paper.

Symbol	Meaning
d	Effective distance
$d_{0}$	Reference distance
n	Path loss factor
Ψ	Gaussian random variable
ρ	Vehicle density
λ	Poisson arrival process parameter
$B_{v}$	Data offloading by a vehicle
$B_{RSU}$	Bandwidth of roadside unit
$\bar{s}$	Average speed of the vehicle
*r*	Radius of RSU’s coverage area
*D*	Diameter of RSU’s coverage area
$d_{ac}$	Distance between two consecutive RSUs with no wireless coverage
*B*	Core network bandwidth
$D_{Vol}$	Data volume
μ	Probability of vehicles to participate
$E_{inc}$	Incremental Energy cost

**Table 3:** Energy Cost per link in MJ/TB.

From	To	Cost/TB	Distance (Km)	Speed (Km/h)
1	2	0.96	40	50
1	3	2.41	100	70
5	3	0.24	10	50
5	6	1.45	60	60
3	4	1.2	50	50
4	2	2.18	90	80
4	6	0.48	20	50
7	1	2.53	105	100
7	5	1.81	75	80
2	8	2.30	95	80
6	8	1.57	65	60

**Table 4:** Core network cost to transfer 1 TB on each link.

From	To	Lan L3 Switches	Edge Routers	Core Routers	Up BW	Down BW	MJ/TB
1	2	9	2	15	0.1	0.1	5.095
1	3	6	2	3	0.1	10.0	4.355
5	3	8	2	14	1.0	1.0	2.03
5	6	11	2	3	1.0	1.0	2.275
3	4	6	2	5	0.1	1.0	4.4
4	2	9	2	6	1.0	10.0	2.02
4	6	8	2	6	0.1	0.1	4.74
7	1	11	2	7	1.0	1.0	2.36
7	5	6	2	7	0.1	0.1	4.44
2	8	11	2	5	1.0	1.0	2.32
6	8	13	2	14	0.1	0.1	5.71
1	6	8	2	9	1.0	10.0	1.925
2	6	14	2	6	1.0	1.0	2.635
3	6	9	2	15	1.0	1.0	2.215
4	8	11	2	2	0.1	0.1	5.135
